# The mechanisms of granulation of activated sludge in wastewater treatment, its optimization, and impact on effluent quality

**DOI:** 10.1007/s00253-018-8990-9

**Published:** 2018-04-28

**Authors:** Britt-Marie Wilén, Raquel Liébana, Frank Persson, Oskar Modin, Malte Hermansson

**Affiliations:** 1 Division of Water Environment Technology, Department of Architecture and Civil and Engineering, Chalmers University of Technology, SE-412 96 Gothenburg, Sweden; 2 Department of Chemistry and Molecular Biology, University of Gothenburg, SE-405 30 Gothenburg, Sweden

**Keywords:** Aerobic granular sludge, Granule stability, Microbial community composition, Wash-out dynamics, Process performance, Granulation mechanisms

## Abstract

Granular activated sludge has gained increasing interest due to its potential in treating wastewater in a compact and efficient way. It is well-established that activated sludge can form granules under certain environmental conditions such as batch-wise operation with feast-famine feeding, high hydrodynamic shear forces, and short settling time which select for dense microbial aggregates. Aerobic granules with stable structure and functionality have been obtained with a range of different wastewaters seeded with different sources of sludge at different operational conditions, but the microbial communities developed differed substantially. In spite of this, granule instability occurs. In this review, the available literature on the mechanisms involved in granulation and how it affects the effluent quality is assessed with special attention given to the microbial interactions involved. To be able to optimize the process further, more knowledge is needed regarding the influence of microbial communities and their metabolism on granule stability and functionality. Studies performed at conditions similar to full-scale such as fluctuation in organic loading rate, hydrodynamic conditions, temperature, incoming particles, and feed water microorganisms need further investigations.

## Introduction

Many wastewater treatment plants need capacity extension due to stricter treatment demands. At the same time, surface area is often limited. Especially the nitrification step of traditional nitrogen removal requires large reactor volumes when based on the conventional activated sludge process due to the long solids retention time (SRT) needed to allow for slow growing bacteria. Also, solids-liquid separation problems associated with filamentous bacteria or poor floc formation are common in conventional activated sludge processes and require plant extensions. To overcome these problems, new more compact and efficient treatment technologies have been developed for nutrient removal during the last decades such as integrated fixed film activated sludge (IFAS), membrane bioreactors (MBRs), moving bed biofilm reactors (MBBR), and aerobic granular sludge (AGS).

AGS for wastewater treatment has gained increasing interest due to its advantages compared to conventional activated sludge: excellent settling properties which enables high suspended solids concentrations in the aeration tank and operation at shorter hydraulic retention times (HRTs). Since the middle of the 1990s when the first laboratory-scale sequencing batch reactors (SBRs) were applied (Mishima and Nakamura 1991; Morgenroth et al. 1997), several studies followed which showed that granules were relatively easy to obtain, and that these had good removal efficiency for organic material (Beun et al. 1999), nitrogen (Beun et al. 2001; Dangcong et al. 1999), and phosphorus (de Kreuk and van Loosdrecht 2005). Since AGS is mainly applied in SBRs, secondary settlers are not needed (Morgenroth et al. 1997). The term *aerobic* granular sludge comes from the first systems that were operated entirely at aerobic conditions, whereas nowadays, various redox conditions (aerobic, anoxic, and anaerobic) are applied to efficiently remove organic matter and nutrients using granular sludge. Granulation starts to occur under certain environmental conditions, namely batch-wise operation with feast-famine feeding, high hydrodynamic shear forces, large height to diameter ratio of the reactor, and short settling time to select for dense microbial aggregates. The large aggregate size of AGS makes simultaneous nitrification, denitrification, and phosphorus removal possible in one reactor due to large diffusion gradients of electron donors and acceptors, creating different redox conditions, within the granule. This enables growth of different guilds of microorganisms in different parts of the granule (de Kreuk and van Loosdrecht 2005; Szabó et al. 2017a). AGS has been cultured from different inoculums using synthetic, domestic, and industrial wastewaters under different reactor conditions. Generally, laboratory-scale reactors with synthetic wastewater gives stable granules within a few weeks, or even faster, e.g., (Szabó et al. 2016), whereas pilot-scale and full-scale reactors require longer start-up periods and granule instability is common. There are today few documented full-scale applications (Li et al. 2014a; Liu et al. 2017; Pronk et al. 2015; Świątczak and Cydzik-Kwiatkowska 2018).

Just as for the conventional activated sludge process, stable aggregation of the granule biomass is important to achieve low concentrations of suspended solids in the effluent, but also for efficient nutrient removal. Even though full-scale applications exist, and several studies have been made to assess which operational parameters are critical to optimize the granulation, the underlying mechanisms behind granulation are far from understood.

During the last years, a number of review papers have been published about the AGS technology, e.g., (Adav et al. 2008c; de Kreuk et al. 2007a; Franca et al. 2018; Gao et al. 2011a; Khan et al. 2013; Lee et al. 2010; Liu et al. 2009; Liu and Tay 2002; Maszenan et al. 2011; Nancharaiah and Kiran Kumar Reddy 2018; Sarma et al. 2017; Seviour et al. 2012b; Show et al. 2012; Winkler et al. 2018; Zhang et al. 2016). The focus of these reviews has been mainly on operational factors that influence the granulation process, the role of extracellular polymeric substances (EPS), physical and chemical aspects of granule stability, and carbon and nutrient removal, as well as treatment of recalcitrant compounds. In most reviews, little attention is given to the influence of the microbial community on the mechanisms involved in the granulation process. The development of new molecular methods has made it possible to identify the microbial community at a high resolution. The aim of this review is to assess the available literature on the mechanisms of aerobic granulation with special attention given to the microbial interactions involved.

## Sludge granulation

**Fig. 1:**
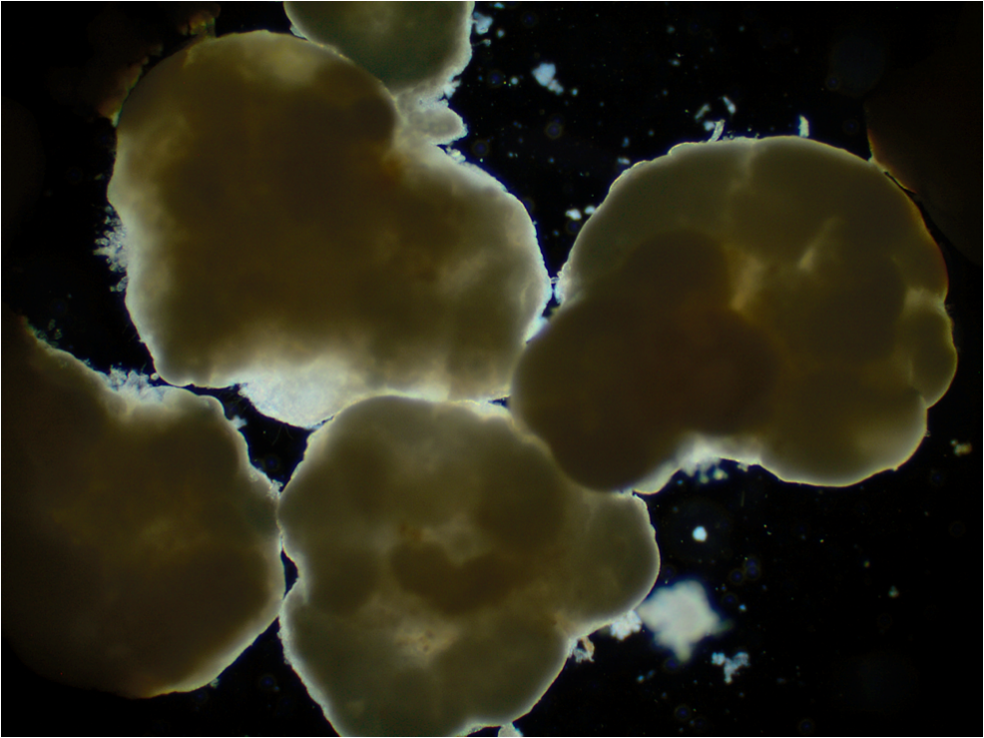
Light microscopy image of aerobic granular sludge

Granulation of the sludge results from biotic and abiotic interactions between microorganisms and sludge particles, resulting in the development of very compact spherical-shaped aggregates with a diameter of approximately 1–3 mm (Fig. 1) where the microbial cells are self-immobilized in a matrix of EPS. Several granulation mechanisms have been proposed but no consensus has been achieved. However, these mechanisms do not necessarily act alone but instead, they are most likely simultaneously involved in the granulation, influencing the processes differently. Granulation has been described to occur in several steps (Liu and Tay 2002) including (1) cell-to-cell contact, (2) attractive forces between cells causing them to aggregate, (3) maturation of the microbial aggregates by forming a matrix of EPS onto which cells can attach and multiply, and (4) formation of a three-dimensional structure shaped by hydrodynamic forces and the microorganisms involved. Filamentous fungi and stalked protozoa have been reported to be important for the granular structure conformation, increasing the surface where the bacteria can attach (Beun et al. 1999; Weber et al. 2007). Granulation has also been described as a consequence of a dynamic floc/particle aggregation and breakage (Verawaty et al. 2012; Zhou et al. 2014) or microcolony outgrowth (Barr et al. 2010a). However, each step in the formation of granules is complex and is influenced by different physical, chemical, and cellular mechanisms.

### Initial stages of the granulation process

The initial stages of the granulation of sludge is determined by different forces and properties of the biomass including hydrodynamic forces, diffusion, cell mobility, and cell surface properties (Liu et al. 2009; Liu and Tay 2002).

#### Cell-cell contact and micro-aggregation

Cell-to-cell contact and aggregate formation are highly dependent on the ability that microorganisms exhibit to develop aggregates, which are determined by cellular mechanisms and their physical and chemical characteristics (Liu and Tay 2002). Cell surface hydrophobicity is an important factor for granule development initiation. During granulation, the biomass tends to become increasingly hydrophobic (Liu et al. 2003; Show et al. 2012). An increase in the protein/polysaccharide ratio would decrease the negative surface charge of granules, which should lead to reduced electrostatic repulsion between bacterial cells and thereby enhance granulation (Gao et al. 2011a; Zhang et al. 2007). However, contrary to this idea, lower protein/polysaccharide ratio has been reported during granule formation (Yuan et al. 2017). The increase in hydrophobicity seems to be due to a higher protein/polysaccharide ratio, which is caused by changes both in EPS and bacterial community composition (Guo et al. 2011; McSwain et al. 2005). The changes in the cell facilitate bacteria to aggregate (Liu and Tay 2002). Wan et al. (2015) proposed that negatively charged bacteria attach to an inorganic core of calcium and phosphate precipitates and produces exopolysaccharides promoting the microbial aggregation. The cell aggregation in granules seems to follow the same colloidal interactions and mechanisms as in activated sludge (but granules can withstand stronger shear forces) and are influenced by the same multitude of factors, including but not limited to DLVO-type interactions (Hermansson 1999), bridging of EPS by cations (Bruus et al. 1992), hydrophobic interactions (Olofsson et al. 1998; Urbain et al. 1993), cell surface charge (Zita and Hermansson 1997), and surface tension of the water phase (Olofsson et al. 1998).

#### EPS production

It is well-established that EPS are important for the long-term stability of aerobic granules (Adav et al. 2008d) but contradictory results can be found in the literature regarding the role of EPS in granulation and the operational parameters that influence its production. Due to its compact structure, extraction of EPS from granules is more difficult than from activated sludge, and this together with the various extraction and analytical methods that have been used give different conclusions regarding the role of EPS in granulation (Adav and Lee 2008a; McSwain et al. 2005). Adav et al. (2008d) studied the roles of individual components of EPS on the structural stability of phenol-fed granules and found that selective enzymatic hydrolysis of extracellular proteins, lipids, and α-polysaccharides had little effect, whereas granule disintegration occurred when β-polysaccharides were hydrolyzed. Exopolysaccharides or glycosides have been found to be gelling agents in aerobic granules, distinctly more adhesive than EPS in activated sludge (Seviour et al. 2009). Lin et al. (2010) extracted alginate-like exopolysaccharides from aerobic granules cultivated in a pilot plant. The exopolysaccharides showed gel-forming properties in the presence of calcium chloride and were suggested to contribute significantly to the hydrophobicity and the elastic structure of the granules. To be able to understand the mechanisms of granulation, methods for characterization of these exopolysaccharides must be further developed to be able to identify the individual polymers and their interactions within the aggregate (Seviour et al. 2012a). Caudan et al. (2014) showed that, by incubating aerobic granules with two different enzymes under the exposure of shear stress, α (1–4) glucans and proteins are key polymers for granule formation and divalent cations play a bridging role. Little is known about the biosynthesis of these compounds by different bacterial species.

#### Cell-to-cell communication

An important mechanism involved in the granulation is quorum sensing (QS) (Zhang and Li 2016). QS regulates many different functions in bacteria (Wang et al. 2017). Several QS regulation systems are involved in biofilm development, such as EPS production (Decho et al. 2010; Nadell et al. 2009). Recent studies have shown the importance of QS for granulation and granular stability (Jiang and Liu 2012; Liu et al. 2016a; Wan et al. 2013). For instance, the concentration of acyl-homoserine lactones (AHL), a common autoinducer in Gram-negative bacteria, increases with granulation (Jiang and Liu 2012) and granular sludge was shown to have a higher content of AHLs than floccular sludge (Li et al. 2014c; Lv et al. 2014c). A higher QS activity during granulation seems to be connected with a higher production of gel-forming EPS, with higher hydrophobicity, involved in increased aggregation and stability of granules (Li and Zhu 2014; Li et al. 2014b). Furthermore, when an SBR was inoculated with floccular sludge and fed with synthetic wastewater, a strong positive correlation of EPS production, community composition changes, and AHL concentrations was seen, whereas a correlation between granular disintegration and reduction of AHL content was found (Tan et al. 2014). Also, several studies have shown that a higher QS activity was linked to a higher microbial attachment potential (Lv et al. 2014a, b, c). Attachment of suspended bacteria was higher when supernatants of mature granules, compared with flocs, were used, presumably because of higher autoinducer concentrations in the former (Ren et al. 2010). Granulation of nitrifying sludge dominated by *Nitrosomonas* could be enhanced by adding exogenous AHLs which increased the extracellular proteins and autotrophic biomass (Wu et al. 2017).

The inhibition of QS, quorum quenching (QQ), has also been found to have an important impact on granulation. QQ was an important modulator of QS during granulation in an SBR inoculated with activated sludge treating synthetic wastewater (Tan et al. 2015). In fact, a shift in the community was observed during granulation, with a higher proportion of QQ active bacteria in the floccular sludge and a higher proportion of bacteria with QS activity in the granules. Addition of the enzyme Proteinase K led to hydrolysis of extracellular proteins and a subsequent granule dispersion. This was presumably due to collapse of the EPS matrix, at the same time as the concentrations of AHL and autoinducer-2 (AI-2) decreased, probably due to inhibition of the of the quorum sensing receptor proteins by proteinase K (Xiong and Liu 2013). In another study, inhibition of the ATP generation was found to reduce the production of AHL and AI-2 and reduced the amount of EPS which led to smaller and less compact granules (Jiang and Liu 2013). The second messenger, cyclic diguanylate (c-di-GMP) is widely used by bacteria to regulate the production of exopolysaccharides, and can hence also affect granulation. Wan et al. (2013) demonstrated that by adding Mn^2+^ ions, which interfere with the c-di-GMP, granules disintegrated as a result of decreased intracellular concentration of c-di-GMP and thereby decreased extracellular concentrations of polysaccharides and proteins. As a consequence, typical polysaccharide producers, *Acinetobacter* sp., *Thauera* sp., *Bdellovibrio* sp., and *Paracoccus* sp. were washed out from the reactors.

### Later stages of granulation

The AGS reactor conditions enhance the aggregation of microorganism shaping the young granules selecting for regular, round, dense, and compact aggregates (Liu and Tay 2004). Once the microbial aggregates have developed, they grow in size and different ecological niches are created by the substrate gradients in the granule, allowing the coexistence of a diverse community with multiple functions utilized in wastewater treatment (Winkler et al. 2013).

#### Microbial composition of granules

Microbial selection is triggered by operational parameters such as type of substrate, organic loading rate (OLR), food-to-microorganism (F/M) ratio, COD/N ratio, solids retention time, settling time, and redox conditions. It has been claimed that AGS has a higher microbial diversity than floccular sludge since they provide more ecological niches due to the substrate gradients created within the aggregate. Despite this, the same functional groups of microorganisms are present in granular and floccular sludge, but with differences in the proportions between phylogenetic groups at a phylum or class level (Guo et al. 2011; Winkler et al. 2013). When assessing the microbial community structure in AGS systems, it is important from which type of treatment configuration the data is obtained; AGS systems are generally applied for COD, COD and N or simultaneous COD, N, and P removal. Liu et al. (2010a) inoculated a pilot-plant fed with real wastewater and operated with COD and N removal, with activated sludge and observed that it took 400 days to obtain granules, still with some floccular sludge present. The microbial community structure, measured with DGGE, was very similar for the granules and the floccular sludge indicating that there was no strong microbial selection for certain groups of microorganisms forming granules. Similarly, Zhou et al. (2014) studied the microbial community in an AGS reactor operated with COD removal fed with real wastewater and found no large difference in microbial community between granules, floccular sludge, or even seeding sludge, with the majority of the bacteria belonging to the phyla *β-Proteobacteria*, *Sphingobacteria*, and *Flavobacteria*. He et al. (2016a) operated an AGS reactor with simultaneous COD, N, and P removal at low ORL fed with synthetic wastewater. The microbial community, analyzed by Illumina MiSeq sequencing, showed a fast change in microbial diversity and richness during granulation with *Proteobacteria*, *Firmicutes*, *Bacteroidetes*, *Chloroflexi*, and *Actinobacteria* as the most abundant bacteria at phylum level. Illumina sequencing was used by Aqeel et al. (2016) to investigate the dynamics in microbial composition during granulation in laboratory-scale reactors fed with synthetic wastewater for COD removal. Both microbial diversity and richness decreased during granulation with the genera *Rhodanobacter* dominating at the maturation stage of granulation which coincided with the increase in protein content of the EPS. Fan et al. (2018) compared granulation in identical reactors operated either with domestic wastewater or synthetic wastewater with the same COD and N load. Illumina sequencing showed a sharp decrease in bacterial diversity during the granulation with a different bacterial community structure in the granules compared to the seed sludge. Surprisingly, the microbial community was similar for the two reactors where the minor genera in the seed sludge *Arcobacter*, *Aeromonas*, *Flavobacterium*, and *Acinetobacter* became dominant in the granules and ammonium-oxidizing archaea (AOA) were gradually washed out, whereas ammonium-oxidizing bacteria (AOBs) and nitrite-oxidizing bacteria (NOBs) were retained. The microbial community structure, analyzed by Illumina, in a full-scale plant performing COD, nitrogen, and phosphorus removal showed that the abundance of *β*-*proteobacteria*, δ-*Proteobacteria*, *Flavobacteria*, and *Cytophagia* increased in abundance when converting activated sludge into AGS (Świątczak and Cydzik-Kwiatkowska 2018). Granules and flocs from a full-scale treatment plant designed for COD and N removal receiving both industrial and domestic wastewater were assessed by 454 pyrosequencing (Liu et al. 2017). Even though the granules and flocs were cultivated in the same reactor, their biodiversity and richness were different. The granules contained mainly the phylum *Planctomycetes*, *Proteoobacteria*, and *Bacterioidetes* and the archaea *Euryarchaeota*, whereas the flocs were dominated by *Proteobacteria*, *Bacterioidetes*, and *Planctomycetes* and the archaea in flocs were mainly *Methanosaeta.* The microbial community development has been found to differ when different recalcitrant organic compounds have been supplied as carbon source (Maszenan et al. 2011).

#### Granule size increase and microbial stratification within the granules

**Fig. 2:**
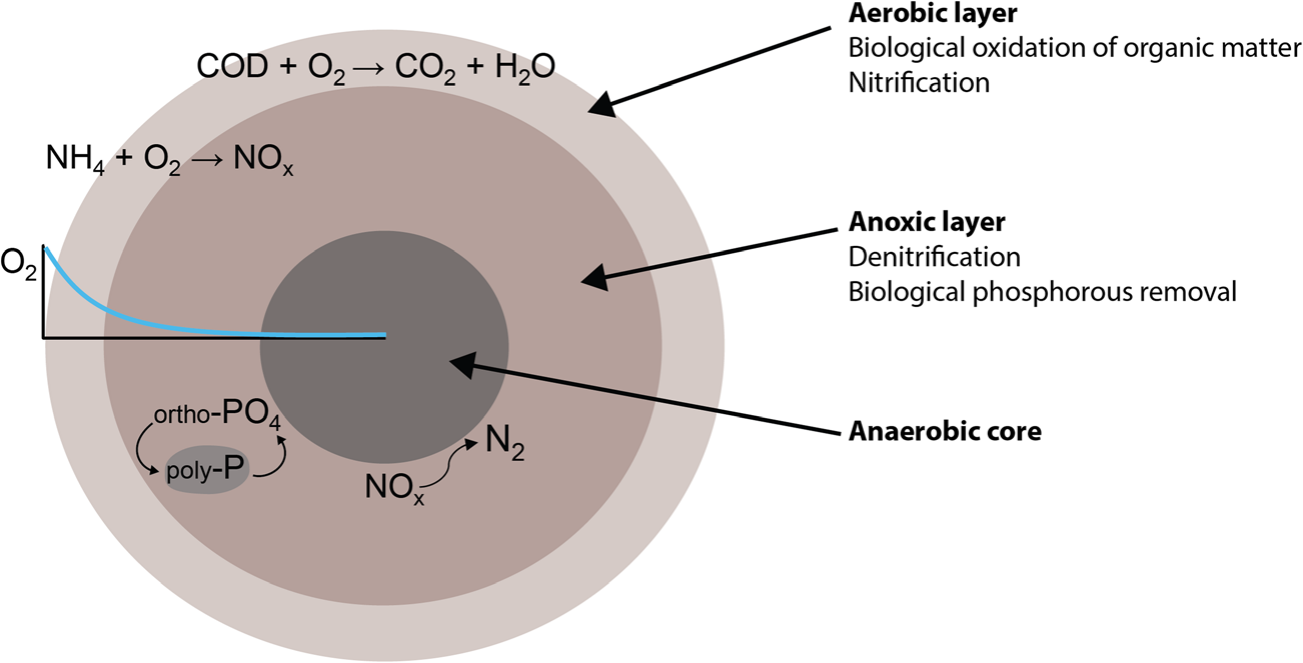
Schematic drawing of an aerobic granule with the different conversion processes for organic material, nitrogen, and phosphorus, taking place within the different redox zones

The development in granule size is dependent on a complex interaction of different environmental parameters and is relatively uncontrollable. Granules seem to reach a certain more or less stable granule size determined by the balance between granule growth, attrition, and breakage, which is a consequence of the process conditions such as shear (Verawaty et al. 2013). Microorganisms grow both at the surface and inside the granule and this will lead to differences in size and structure. Bacteria are not equally distributed within the granules as some are more abundant at the outer layers of the granule and some in the interior (de Kreuk and van Loosdrecht 2005). Conceptual and mathematical models often simplify the granular structure by considering granules as a multilayer sphere with decreasing oxygen and substrate gradients from the outside to the core of the granule (Fig. 2). Hence, according to these models and in general terms, nitrifiers are located in the oxygen penetrated outer layers, whereas denitrifiers and phosphate-accumulating organisms (PAOs) are located in the inner layers (Winkler et al. 2013; Xavier et al. 2007). Guimarães et al. (2017) showed that a microbial stratification existed in granules operated with COD and nitrogen removal fed with low strength real wastewater. AOBs dominated the outer layer, whereas NOBs and denitrifiers were located in the inner parts. Lv et al. (2014c) showed that mature granules performing COD and N removal had a core with *Rhodocyclaceae*, but that the core was covered by an outer shell containing both aerobic and anaerobic strains; in the beginning of the granulation, *Flavobacteriaceae*, *Xanthomonadaceae*, *Rhodobacteraceae*, and *Microbacteriaceaea* were abundant, with time anaerobic strains becoming more abundant. Furthermore, using fluorescence in situ hybridization (FISH) and confocal laser scanning microscopy (CLSM), Szabó et al. (2017b) revealed that nitrifiers grew both at the oxygen-rich surface but also inside the granule in channels and voids. The presence of AOBs in the inner locations of the granules indicates that both oxygen and ammonia are transported across the granule through the channels. These results were observed for three different organic loading rates. It is well known that biofilms are heterogeneous structures and in most of the cases they contain pores, channels, mushroom-like structures, and water-filled voids (Flemming and Wingender 2010; Wimpenny et al. 2000) and, therefore, it is no surprising that aerobic granules would also show high structural heterogeneity. By applying microscopy in combination with fluorescence staining techniques and image analysis, complex three-dimensional structures of granules were demonstrated and it was proposed that granules grow from the inside by outgrowth to form granules rather than aggregation of small microbial colonies (Gonzalez-Gil and Holliger 2014).

It is assumed that bacteria growing at the surface of granules are more easily detached due to shear forces compared to the ones further in. Winkler et al. (2012) evaluated the SRT for different bacterial groups in AGS and floccular sludge and determined a dimensionless wash-out ratio for specific bacteria. Here, nitrifiers were present in the outer oxic layers and had a SRT of 11 ± 3 days; PAOs and glycogen-accumulating organisms (GAOs) were found both at the outer parts and further in and had a longer SRT (13 ± 4 days), whereas Archaea were only present in the inner part. By comparing the community composition by high-throughput amplicon sequencing in both the AGS and effluent phase, Szabó et al. (2017a) also found differences in wash-out dynamics for different bacteria. The microbial community in the washed-out biomass was similar but not identical to the granular biomass; certain taxa such as *Flavobacterium* spp. and *Bdellovibrio* spp. were relatively more abundant in the granules compared to the effluent, whereas other taxa such as *Meganema* sp. and *Zoogloea* sp. had relative lower abundance in the granules compared to the effluent.

### Selection forces behind granulation

Despite the well-established method of transforming activated sludge into granules, it is a complex process and the ecological mechanisms are largely unknown. Granules are generally thought to be obtained by (1) applying high hydrodynamic shear forces, (2) feast-famine alternation, and (3) washing-out of the non-granulated biomass (Adav et al. 2008c; de Kreuk and van Loosdrecht 2004; Lee et al. 2010; Liu and Tay 2004; Show et al. 2012).

Generally speaking, bacteria can exist both in a planktonic or attached mode and biofilm/granule development is key for retention of prokaryotes in flowing environments that develop under shear forces (Boltz et al. 2017). Thus, granulation is a response to specific selection pressures. High shear forces stimulate bacteria to increase the production of extracellular polymers with a higher polysaccharide/protein ratio, increasing the hydrophobicity of the biomass (Tay et al. 2001). Therefore, high shear force assists the formation of compact and denser aerobic granules shaping the granules into rounded aggregates by removing outgrowing structures. Feast-famine alternation and anaerobic feeding increases bacterial cell surface hydrophobicity, accelerate the microbial aggregation, and promote the growth of slow growers (Adav et al. 2008c; Liu et al. 2004).

Washing out the non-granulated biomass is considered an important selection force for sludge granulation. But according to the results from various studies, high wash-out rates would act as an accelerant of granulation by the physical selection of bigger particles. Indeed, in our laboratory (results not published), and in others, granulation has been observed even at long settling times with a low degree of wash-out of suspended matter (Barr et al. 2010a; Dangcong et al. 1999; Dulekgurgen et al. 2003; Weissbrodt et al. 2013b), but as expected, much longer reactor run times were needed to obtain aerobic granules under these circumstances. Higher shear forces have been found necessary to achieve granulation when long settling times is applied (Chen and Lee 2015; Zhou et al. 2014). Moreover, Szabó et al. (2017a) showed that during the initial stages of granulation in a system for COD and nitrogen removal, most genera were washed out proportionally to their relative abundance on the floc-particles. Therefore, the biomass was proportionally washed out until granules emerged. Once granules emerged, microorganisms located on the granular surface where preferentially washed out from the reactors due to erosion of the granules while those growing in the granular interior were retained in the reactor. Some bacteria retained in the reactors still displayed a decreasing trend of relative abundance, indicating that they were retained during the physical particle selection but were thereafter outcompeted by better adapted other ones. Zhou et al. (2014) observed that when flocs and crushed granules were differently labeled with fluorescent microspheres and mixed in a reactor, flocs detach and re-attach to granules in a random manner. This indicates that floccular sludge is not washed out from the reactor due to the inability of certain microorganisms to form granules, instead microorganisms move between granular and floccular sludge randomly (Zhou et al. 2014; Verawaty et al. 2012). These results indicate that high wash-out dynamics is not a requisite for granulation and therefore should not be considered as an important selection pressure for sludge granulation.

### Predation and granulation

Predation is one of the most important interactions between living organisms, as a major cause of bacterial mortality with direct implications on the genetic and functional structure of the community (Jousset 2012). Bacteriophages (virus), predatory bacteria, and protists are the most important microbial predators (Johnke et al. 2014). Predation has been reported to have important implications in the process performance. Bacteriophages have been reported to display 10 to 100 times higher diversities than bacteria in aquatic ecosystems and to be responsible up to 71% of the bacterial mortality (Johnke et al. 2014). Barr et al. (2010b) operated a laboratory-scale SBR for enhanced biological phosphorous removal. They associated an unexpected drop in phosphate-removal performance with bacteriophages infection of the key phosphate-accumulating bacterium in the reactor due to the presence of elevated levels of virus-like particles in the reactor. Moreover, the addition of bacteriophage-rich supernatant to other reactors affected negatively the phosphate removal performance. Predatory bacteria feed on other microbial cells and they have been found in a variety of environments (Martin 2002). The presence of predatory bacteria in aerobic granules and its persistence during granulation has been reported (Li et al. 2014d; Szabó et al. 2017a; Wan et al. 2014; Weissbrodt et al. 2014). Predatory bacteria and their effect on microbial populations is, however, poorly understood. It is believed that they have an important influence on microbial community structure and dynamics. For instance, it has been reported that the predation of *Nitrospira* sp. by *Micavibrio*-like bacteria can have a direct impact on the nitrification process (Dolinšek et al. 2013). Stalked ciliates have been observed in higher numbers growing on the granule surface (Lemaire et al. 2008a). Winkler et al. (2012) reported *Vorticella*-like protist actively grazing on bacteria in aerobic granules. Protist grazing activity induces different phenotypes of bacteria, such as biofilm development, as a survival strategy (Matz and Kjelleberg 2005). A higher biofilm production and aggregation has been reported due to the grazing activity of protists (Liébana et al. 2016; Matz et al. 2004). Predation by protists can also cause a reduction of the biofilm bacteria (Huws et al. 2005) and even extend to deep biofilm layers (Suarez et al. 2015). Therefore, it is reasonable that protists, and also predatory bacteria and bacteriophages, have a direct impact on granulation and granule structure.

## Process configurations and operational conditions affecting granulation

**Fig. 3:**
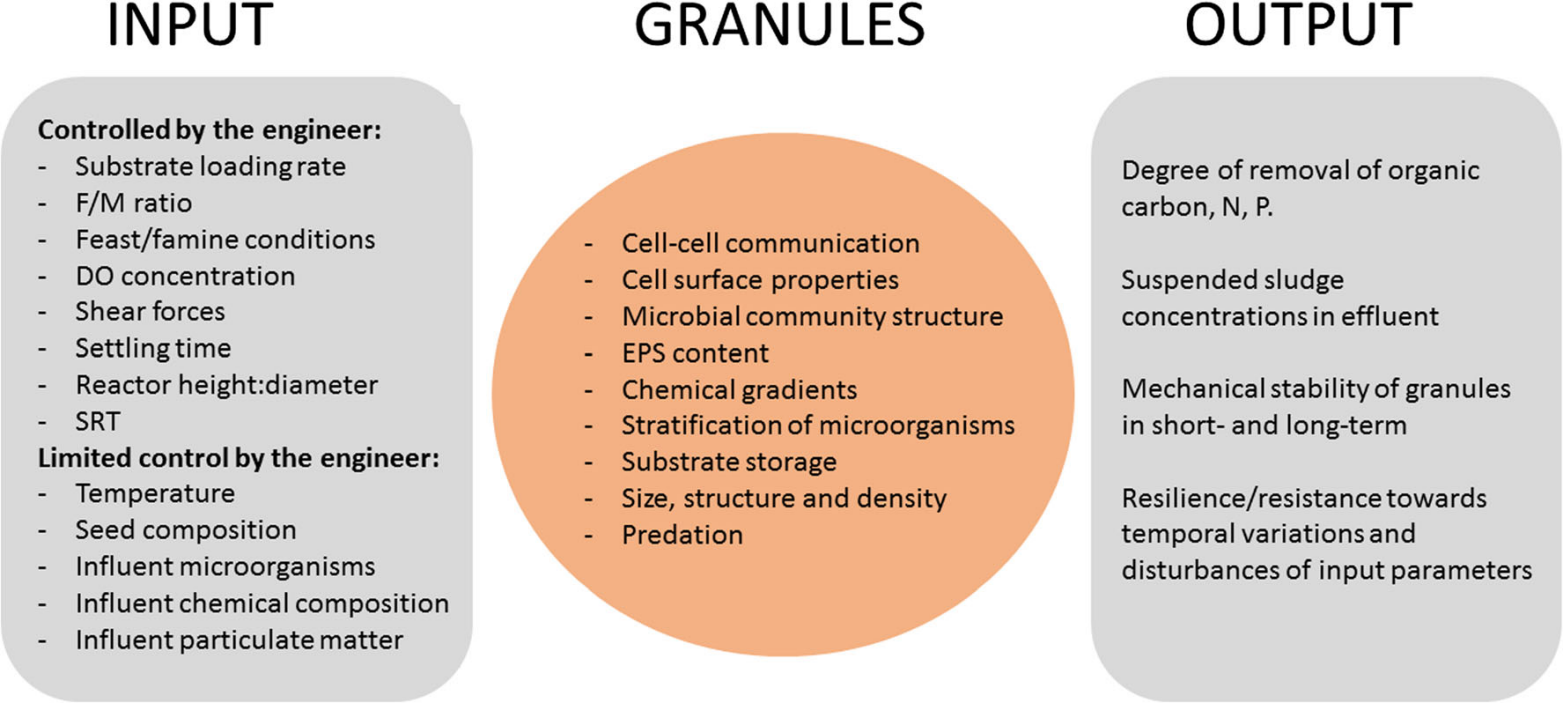
Schematic illustration of the various process parameters that effect the granule formation and the effect on effluent

Several operational and environmental factors are known to influence the formation and stability of granules. Some of the factors such as hydrodynamic shear forces, settling time, volume exchange ratio, and organic loading rate can be controlled by the treatment process designer or operator. Other factors, such as temperature and composition of the influent wastewater, are more difficult to control (Fig. 3).

### Hydrodynamic shear forces

Shear forces influence the shape and structure of the aerobic granules. Most AGS laboratory-scale reactors are designed as bubble columns where shear forces are created by the aeration rate, expressed as up-flow superficial velocity, typically in the range 1–2 cm s^−1^. In earlier studies, it was found that granules would not form at superficial air velocities lower than 1.2 cm s^−1^ (Beun et al. 1999; Tay et al. 2001). Adav et al. (2007) found that high aeration rate accelerated granule formation with phenol as carbon source and formed smaller granules compared to intermediate aeration rate and that they contained higher quantities of EPS with higher protein/polysaccharide ratio which contradicts the findings by Tay et al. (2001). Liu and Tay (2006) obtained stable granules when the aeration rate was reduced to 0.55 cm s^−1^ during the famine phase when the growth rate and oxygen consumption is low. More recent research show that granules form at superficial air velocities of 0.8 cm s^−1^ but with more porous and unstable structure (Lochmatter and Holliger 2014). Granules were also formed at velocities as low as 0.42 cm s^−1^ during low strength (300 mg COD L^−1^) wastewater treatment, but not with medium or high strength wastewaters (600 or 1200 mg COD L^−1^) (Devlin et al. 2017). He et al. (2017) operated a system for simultaneous nitrogen and phosphorous removal at even lower superficial gas velocities, ranging from 0.04 to 0.17 cm s^−1^, at an OLR of 0.3 kg COD m^−3^day^−1^. At a low superficial gas velocity, the granules showed poorer settling properties with decreased protein/polysaccharide ratio but an increased rate of nitrogen removal was measured. The microbial analysis revealed an increase of AOB and PAOs, while GAOs and NOBs decreased in numbers (He et al. 2017). Successful granulation has been obtained in SBRs with completely stirred tank configuration due to growth of PAOs and GAOs, but it took three times longer for the granules to form compared to in bubble columns (Weissbrodt et al. 2013a). Devlin et al. (2017) proposed that granulation is dependent on various parameters that selects out fast growing aerobic microorganisms; at higher OLR, more shear is needed to shear off the fast growing microorganisms at the surface, whereas at low OLR, less shear is required. Hence, they concluded that shear force is not a requirement for granules to form but shear mitigates surface fouling when organic loads exceed the capacity for anaerobic uptake.

### Settling velocity and exchange ratio

It has been stressed in many studies that settling time is an important parameter when selecting for granules. High exchange ratio with a short decanting phase selects for granules in the reactor and leads to faster granulation (Nancharaiah and Kiran Kumar Reddy 2018; Sheng et al. 2010). Gao et al. (2011b) compared different strategies to enhance AGS formation and found that the fastest granulation was obtained by shortening the settling time from 15 to 5 min in 11 days. Ni et al. (2009) operated and achieved successful granulation in a pilot-scale reactor fed with low-strength real wastewater at a settling time of 15–30 min and an exchange ratio of 50–75%. Liu et al. (2016b) achieved fast granulation by immediately shortening the settling time to 2 min at a high OLR (8 kg m^−3^day^−1^). Already after 24 h, granules started to appear in the three reactors operated at the same OLR but at different HRTs ranging from 8 to 4 h by adjusting the concentration of COD in the inflow. The microbial community structure (measured by T-RFLP) showed a fast shift during the first days after which it stabilized and there was little difference between the three reactors, indicating that the settling time was the strongest microbial selection force. In full-scale AGS plants, settling time is for practical reasons longer, around 30 min, which gives a mixture of granules and floccular sludge (Pronk et al. 2015). In a previous study, selective removal of sludge was applied; a fraction consisting of both very light and very dense granules was removed to retain a wider size range of granules in the reactor (Henriet et al. 2016). This lead to an improved phosphorus removal, since very large and dense granules which are saturated with phosphorus and cannot take up more were removed. Nitrogen and COD removal was maintained with an accumulation of Ca. *Accumulibacter*.

### Organic loading rate

Li et al. (2008) observed that in glucose-fed AGS reactors, the granules had different morphology and contained different microbial species when operated at different organic loading rate (OLR); high OLR (4.5 kg m^−3^day^−1^) resulted in faster granulation, larger and more porous granules compared to lower OLRs (1.5 and 3 kg m^−3^day^−1^). Furthermore, a higher OLR gave a lower microbial diversity measured by DGGE. Schwarzenbeck et al. (2005) observed filamentous growth and granule instability in a study treating dairy wastewater at high OLRs. Szabó et al. (2017b) studied the influence of OLR (0.9, 1.9, and 3.7 kg m^−3^day^−1^) in AGS reactors fed with a mixture of synthetic wastewater with acetate as carbon source and reject from dewatering of anaerobically digested sludge. Almost complete organic carbon and ammonium removal was achieved in all reactors, whereas total nitrogen removal was highest (66%) at the highest OLR and 0% at the lowest. A fast divergence in bacterial community composition after start-up was observed with less than 50% similarity after 6 days and below 40% after 84 days. The three reactor microbiomes were dominated by different genera, mainly *Meganema*, *Thauera*, *Paracoccus*, and *Zoogloea*, which have similar ecosystem functions such as EPS formation, PHA storage, and denitrification and only a minor fraction of nitrifiers (< 3%) were present. The steepest decrease in richness was observed at the highest ORL but after granule maturation, all reactors showed similar richness and evenness; 37 and 16% lower than for the seeding sludge, respectively. Starvation also impacts granular properties. Liu et al. (2016b) operated three parallel lab-scale reactors at the same ORL (by varying the COD concentration in the influent between 1250 and 7500 mg L^−1^) at cycle lengths of 3, 5, and 7 h. The QS autoinducer AI-2 increased steadily during the starvation phase, and at long-term operation the granules became more stable containing higher concentrations of AI-2 and had EPS with higher molecular weight. At the genus level, *Proteobacteria*, *Actinobacteria*, and *Verrucomicrobia* dominated in all reactors. Zhang et al. (2017) operated two parallel reactors at alternating OLR and a cycle length of 6 h. After 30 days of stable reactor performance and well-granulated sludge, the OLR in one reactor was changed to a constant value and the cycle length was shortened from 6 to 4 h, giving shorter starvation period, in the other. Granule disintegration was observed in both reactors simultaneously as the EPS concentration, cell adhesion, and concentrations of AI-2 decreased, indicating that alternating OLR and prolonged starvation was important for granule stability.

#### Feeding strategy

Previous studies have shown that applying feast-famine alternation and anaerobic feeding increases bacterial cell surface hydrophobicity, accelerate the microbial aggregation, and create the appropriate substrate and oxygen gradients in the granule (Liu and Tay 2002; Gao et al. 2011b). Feast-famine conditions also promote the internal storage of organic matter (Adav and Lee 2008a; Liu et al. 2004). By storing organic matter, the bacterial growth rate is decreased, slow growing microorganisms are allowed to develop within the granules, and compact granules are obtained (de Kreuk et al. 2007b). The importance of the selection of slow-growing microorganisms for the development of stable granules was also observed in a full-scale WWTP (Pronk et al. 2015). Slow-growing nitrifying bacteria are, not surprisingly, selected at low C/N ratios (Liu et al. 2004). Growth rate can also be controlled by the cycle configuration; if an anaerobic phase is introduced, PAOs will take up organic matter and store it as PHAs. This further promotes the proliferation of slow growing microorganisms (de Kreuk and van Loosdrecht 2004). Sufficient long starvation period, depending on substrate composition and load, after feeding is needed to ensure stability of the granules and to avoid filamentous outgrowth (Franca et al. 2018).

### Inoculum

AGS reactors are in most cases seeded with activated sludge (Chen and Lee 2015). In AGS reactors fed with real wastewater, a continuous seeding of microorganisms occurs which will influence the microbial community, as opposed to laboratory reactors fed with synthetic wastewater where only the original seeding sludge will influence the community. This is important when drawing conclusions from different studies regarding the microbial community composition. The exact role of the seeding sludge in the granule formation and evolution of the microbial AGS community is not completely understood. Adav and Lee (2008b) managed to cultivate granules from a single bacterial strain, *Acinetobacter calcoaceticus* in a reactor fed with phenol*.* Activated sludge sampled at different occasions from the same WWTP did not always result in successful granulation, indicating that the microbial composition of the seeding sludge is important (Ho et al. 2009); however, their experiments were performed in reactors fed with phenol which is a rather specific type of substrate. Song et al. (2010) compared the microbial community development in AGS reactors seeded with either activated sludge from a beer wastewater treatment or from a municipal wastewater treatment. The granules obtained from the beer treatment appeared faster (within 35 days) and became denser and had a more diverse microbial composition than to the ones obtained from the municipal treatment. Weissbrodt et al. (2012) studied the microbial selection by T-RFLP and pyrosequencing during start-up of five parallel AGS reactors fed with synthetic wastewater. Reactors seeded with activated sludge from a plant removing organic material operated at low air flow rate of 1.8 cm s^−1^ or at 30 °C produced slow-settling and fluffy granules with filamentous *Burkholderiales* (*Sphaerotilus* and *Leptothrix genera*) and *Zoogloea* relatives as predominant phylotypes. However, when seeded with activated sludge from a biological nutrient removal plant, or when operated at higher air flow rate (4.0 cm s^−1^), compact and well-settling granules with high abundance of *Rhodocyclales*-affiliated *Zoogloea*, *Dechloromonas*, *Thauera*, and *Rhodocycluss* spp. were formed. The *Rhodocyclales*-affiliated bacteria produce EPS and store PHA at high organic loadings (Seviour et al. 2012b) and may therefore contribute to granulation.

### Dissolved oxygen concentration

AGS can be formed in a wide range of dissolved oxygen concentrations (DO), from as low as 0.7–1.0 up to 2–7 mg L^−1^ (Winkler et al. 2018), but DO lower than 2–5 mg L^−1^ has been reported to lead to granule instability (Hailei et al. 2006; McSwain Sturm and Irvine 2008; Mosquera-Corral et al. 2005). DO is an efficient control parameter for simultaneous removal of organic carbon, nitrogen, and phosphorus due to the strong diffusion limitations in granules where high DO concentrations lead to a larger aerobic fraction and hence increased nitrification rate, whereas lower DO concentrations increase the denitrification rate. By controlling the DO over the cycle in an SBR, high effluent quality can be achieved (Pronk et al. 2015).

### Temperature

AGS processes have been operated successfully at different temperatures (8–30 °C). Start-up at low temperature (8 °C) has been challenging (de Kreuk et al. 2005). At low temperature, the granules become irregularly shaped and instable with low nutrient removal efficiency. It has been suggested that since the microbial activity is low at low temperatures, DO can penetrate to the inner anoxic part which will hamper the denitrification (de Kreuk and van Loosdrecht 2005). Despite this, AGS reactors have been operated with high efficiency of organic carbon, nitrogen, and phosphorus removal at 10–12 °C (Bao et al. 2009; Jiang et al. 2016). Interestingly, seeding an AGS reactor with cold-acclimated activated sludge was a successful strategy to obtain stable granules performing efficient organic carbon and nutrient removal at 7 °C (Gonzalez-Martinez et al. 2017). At higher temperature (20–30 °C), disturbances in the biological phosphorus removal have been observed due to favorable growth conditions for GAOs which outcompetes PAOs (Lopez-Vazquez et al. 2009a), but Ab Halim et al. (2015) managed to operate AGS reactors at 30, 40, and 50 °C with high removal efficiencies of both nitrogen and phosphorus.

### Wastewater composition

Divalent cations, especially Ca^2+^ and Mg^2+^, have been found to improve granule stability by forming bridges between EPS molecules (Li et al. 2009; Ren et al. 2008). Cations can promote aggregation by (i) neutralizing the negative cell surface charge, (ii) polymer bridging of EPS, and (iii) forming precipitates onto which cells can aggregate. Calcium ions can for instance form precipitates with CO_3_^2−^ or by PO_4_^2−^, which act as a nuclei for microbial aggregation (Ren et al. 2008). Gao et al. (2011b) found that higher organic loading rate with added Ca^2+^ gave larger granules with higher polysaccharide-to-protein ratio. Kończak et al. (2014) compared the binding of divalent cations (Ca^2+^ and Mg^2+^) and multivalent cations (Fe^3+^) to EPS in aerobic granules. It was found that granules fed with Ca^2+^ and Mg^2+^ contained more polysaccharide than the Fe^3+^-fed reactor, whereas the protein content was higher in all reactors fed with cations compared to the control without.

Slowly, biodegradable particulate substrate (such as particulate starch) was found to be adsorbed onto AGS after which it was hydrolyzed extracellularly (de Kreuk et al. 2010). The release of soluble substrate caused filaments and outgrowths giving the granules irregular structures. Ultimately, it resulted in reduced COD and nutrient removal efficiency due to limited storage of organic material in the inner part of the granule. Wagner et al. (2015) compared AGS reactors operated with only soluble carbon (acetate and hydrolyzed peptone) and with soluble and particulate carbon (particulate starch). In the presence of particles, the anaerobic phase had to be prolonged to allow for substrate hydrolysis. As a result of constant access to soluble substrate from the hydrolysis, the granules became less stable with a large fraction of floccular sludge, which resulted in increased effluent suspended solids concentrations.

AGS compact structure protect microorganism from inhibitory compounds. This feature enable AGS technology to successfully treat industrial wastewater such as textile wastewater containing toxic azo-dyes (Lourenço et al. 2015), and inhibitory organic compounds, high strength wastewaters such as dairy, brewery, live-stock effluents, and toxic heavy metals (Maszenan et al. 2011; Nancharaiah and Kiran Kumar Reddy 2018).

## Optimization of the AGS process and influence on effluent quality

Most research about AGS has been performed in small and well-controlled laboratory-scale reactors, and it may not be straight forward to extrapolate results to other scales. On the other hand, it is hard to draw conclusions from studies at pilot- and full-scale, because of uncontrolled conditions. Impact of typical full-scale parameters such as fluctuating wastewater composition, variations in flow rates, particles present in real wastewater, and influence of complex carbon source is not well investigated. However, there are different strategies that can be implemented.

### Granular size and structure

Granule size and structure are obviously crucial parameters for an efficient AGS process since they are linked to the function and microbial activity. Liu and Tay (2015) found that granules of size 0.6–1.2 mm were preferred by AOB, whereas NOB preferred larger granules (1.2–1.8 mm). It is not desirable with very large granules as this leads to instability and smaller surface area which gives reduction in both substrate removal rates and biomass growth rates (de Kreuk et al. 2007b; Liu et al. 2005). Dead layers of biomass has been found in granules larger than approximately 800 μm analyzed by FISH, indicating mass transfer limitations inside the granule core which might lead to voids inside the granule and eventually granule break-up when EPS is consumed as an energy source due to substrate limitations (Tay et al. 2002). Larger granules seem to form at higher concentrations of readily biodegradable organic matter concentrations (Rocktäschel et al. 2015; Weissbrodt et al. 2013b). Low ORL (1.5 kg m^−3^ day^−1^) has been found to give smaller and more compact granules than at higher ORL (3–4.5 kg m^−3^ day^−1^) (Li et al. 2008). For granules to be stable at high OLRs, Devlin et al. (2017) proposed that by increasing the shear, by increasing the upflow air superficial velocity in the reactor, surface outgrowth of the granules caused by rapidly growing aerobic microorganisms could be reduced giving a more stable granulation.

### Granular stability

One critical parameter in the operation of AGS processes is the concentration of suspended solids in the effluent: the poorer ability of flocculation, i.e., the ability of granules to clarify the effluent during sedimentation, compared with activated sludge, which may lead to increased effluent suspended solids concentrations. Hence, additional polishing steps such as filtration is needed to meet the effluent standards, especially if strict requirements on phosphorus applies (Schwarzenbeck et al. 2005). Granule stability is crucial to keep the concentration of suspended solids in the effluent low. Microorganisms compete for substrates inside the granule but also for space to grow. The diffusion of substrates and dissolved oxygen creates different zones; aerobic at the outer surface where fast growing heterotrophs and filaments grow and anoxic and anaerobic zones further in toward the core of the granule. Channels that may contribute to a better diffusion may clog due to particles and colloids in the wastewater and due to production of EPS (Zheng and Yu 2007). Starvation and anaerobic conditions in the core of granules lead to endogenous cell respiration and cell lysis which ultimately give hollow cavities and granule disintegration (Franca et al. 2018). Different OLRs give different growth rate of biomass in the reactor and may therefore influence the effluent suspended solids concentration. Li et al. (2008) observed that during the start-up of three AGS reactors operated at different OLRs, the effluent-suspended solids concentration was higher at higher ORL, but as the granules matured, the difference in effluent suspended solids concentration decreased, indicating that the food-to-microorganisms (F/M) ratio plays an important role.

### Reactor configuration

Since de Kreuk and van Loosdrecht (2004) developed the anaerobic feeding through a stagnant granule bed in a column-type reactor, most studies performed have used this process configuration. In practice, very high height-to-diameter (H/D) ratio is unpractical and it is difficult to get plug-flow with the risk of getting inhomogeneous accumulation of carbon in the granule bed (Winkler et al. 2011). When an AGS reactor was changed from a H/D ratio of 9 to 2 after 250 days, the feeding was changed from plug-flow feeding through stagnant granule bed to fast feeding with mixing (Rocktäschel et al. 2013). The sludge concentration went from 20 to 9 g L^−1^ with effluent suspended solids concentrations changing from 0.10 to 0.05 g L^−1^. In spite of the lower sludge concentration, the nitrogen and phosphorus removal rates increased, but the granules became less stable. Henriet et al. (2016) managed to operate an SBR with a H/D ratio of 1.8 by selecting for PAOs by removal of the poorly granulated fraction of the biomass. Recently, AGS in continuous systems has been successfully operated in lab-scale reactors inoculated with activated sludge from a full-scale biological nutrient removal plant (Devlin and Oleszkiewicz 2018). Three step fed mixed anaerobic tanks in series followed by two aerobic tanks were operated with selective wasting of floccular sludge in a settler equipped with mixing. This is a very attractive solution as continuously operated AGS systems enables simpler retrofitting of existing activated sludge plants, but the challenge is to obtain stable granules in long-term operation.

### Nitrogen removal

It has been reported that during start-up of AGS reactors, nitrogen removal may reduce, presumably due to harsh wash-out of biomass which would lead to reduced SRT and leakage of carbon from the anaerobic feeding phase into the aerobic phase. This gives overgrowth of filaments and zoogloeal populations (Weissbrodt et al. 2012). Often it takes 2–4 months to obtain efficient nutrient removal (e.g., de Kreuk and van Loosdrecht 2005; Gonzalez-Gil and Holliger 2011). Slow growing bacteria such as AOB and NOB are sensitive to reduced SRT. During start-up, the microbial community has been reported to change quickly (Li et al. 2008; Liu et al. 2010b). Szabó et al. (2016) demonstrated that a stepwise decrease of the settling time enabled fast granulation and a rapid start-up of the nitrification process and the biomass was dominated by granules after 10 days of operation with almost full nitrification. The bacterial community composition, measured with T-RFLP and qPCR, changed rapidly during the first 21 days with a reduction in richness and evenness, especially for the nitrifying community. Winkler et al. (2013) compared the microbial populations in an AGS pilot-plant and a full-scale activated sludge plant receiving the same wastewater, operated at the same temperature and HRT and observed a difference; the nitrifying granules contained both *Nitrosomonas* and *Nitrosospira*, whereas the dominating AOB in the seeding sludge was *Nitrosomonas.* Even though the microbial communities were dissimilar, they had similar diversity and evenness suggesting that the different communities had similar functionality. To obtain simultaneous nitrification and denitrification (SND), it is important to control DO; it should be high enough to sustain nitrification at the outer layer and low enough to prevent oxygen from penetrating into the deeper anoxic parts of the granule where denitrification can take place. Beun et al. (2001) obtained efficient SND with stable granules at 40% oxygen saturation, whereas Mosquera-Corral et al. (2005) obtained relatively good SND at similar oxygen saturation but experienced granule instability. For an efficient SND process, the organic material has to be taken up and stored as poly-β-hydroxybutyrate for subsequent use as carbon source for denitrifiers (Beun et al. 2001). The COD/N ratio is important for efficient SND since it acts as a strong selection pressure parameter for either enrichment of heterotrophs or nitrifying bacteria. Kocaturk and Erguder (2016) showed that high COD/N values (7.5–30) gave large and fluffy granules due to favoring growth of heterotrophs, whereas low COD/N ratios (2–5) led to small and dense granules containing more slow growing nitrifiers and concluded that a ratio of 7.5 is optimum for stable granules. Liu et al. (2004) investigated the selection for nitrifying bacteria at different COD/N ratios, ranging from 20 to 3. The lower ratios produced smaller and more compact granules enriched in nitrifiers. Wei et al. (2014) investigated different COD/N ratio for ammonium-rich synthetic wastewater and obtained the highest nitrogen removal at a ratio of 9.

### Phosphorous removal

In AGS systems designed for phosphorus removal, it is important to support the growth of PAOs. By selecting for slow-growing microorganisms such as PAOs, the granules will be more stable also at low DO concentrations (de Kreuk and van Loosdrecht 2005). Competition between PAOs and GAOs may occur under certain circumstances such as high temperature (Gonzalez-Gil and Holliger 2011; Weissbrodt et al. 2013b). In activated sludge, it has previously been found that PAOs are abundant at lower temperatures (10 °C), whereas GAOs dominate at higher temperatures (20–30 °C) (Lopez-Vazquez et al. 2009a; Lopez-Vazquez et al. 2009b). Winkler et al. (2011), however, elegantly showed that by selectively removing biomass from the top of the AGS bed after settling, stable phosphorous removal could be obtained at high temperatures (30 °C) since the denser granules at the bottom contained more PAOs, as measured by FISH, whereas the light top fraction was more abundant in GAOs. The carbon source has been found to affect the stability of the phosphorus removal. Gonzalez-Gil and Holliger (2011) showed that during the first 50 days of operation, the propionate-fed reactor was dominated by *Zoogloea*, *Acidovorax*, and *Thiothrix*, whereas the acetate-fed reactor mainly contained *Thiothrix.* When the biological phosphorus removal activity started, the propionate-fed reactor performed well, whereas the acetate-fed reactor was instable. Both reactors contained *Accumulibacter* and *Competibacter*, but also *Chloroflexi* and *Acidivorax*. By using T-RFLP based on the *ppk1* gene (a polyphosphate gene that can be used as a phylogenetic marker), it was found that the two reactors contained different types of *Accumulibacter.*

### Simultaneous biological nutrient removal

Simultaneous biological nutrient removal (SBNR) can be obtained by introducing an anaerobic phase. The phosphorus removal can be further enhanced by introducing a longer anaerobic filling phase (de Kreuk et al. 2010) because during the anaerobic phase, PAOs are enriched in the presence of volatile fatty acids (de Kreuk and van Loosdrecht 2005). de Kreuk and van Loosdrecht (2004) studied the selection of PAOs and GAOs by reducing the DO concentration and prolonging the anaerobic feeding phase. In this way, all acetate was converted to more slowly biodegradable storage polymers. PAOs dominated when fed with phosphorus and GAOs without, but no difference in granule appearance was observed. They concluded that slow growing microorganisms grow at the outer surface and that less shear is needed to form dense aggregates also at low DO, something that is not possible at high ORL and low DO (Mosquera-Corral et al. 2005). Lemaire et al. (2008b) operated lab-scale reactors for SBNR and found that PAO (*Accumulibacter*) grew mainly at the outer oxygenated 200 μm while GAO (*Competibacter*) were more abundant in the anoxic core of the granule. He et al. (2016b) obtained excellent SBNR in an AGS system fed with acetate as carbon source in anaerobic/oxic/anoxic (AOA) mode, which utilize the organic carbon more efficiently.

## Outlook

There is a large number of published research in the field of AGS but still many aspects are not fully understood and require further investigations. The effluent particle concentrations are critical for applying AGS in full-scale. Due to granule instability, fluctuations in effluent suspended solids concentrations occur and strict effluent limits on total phosphorus may therefore be difficult to meet. New technologies such as aerobic granular membrane reactors (AGMBR) could be a solution, where an efficient retention of suspended particles is possible (Liébana et al. 2018). Studies using real wastewater are limited, i.e., variable OLRs, temperature, and composition may cause operational problems. Also, separate investigations of typical “real wastewater issues” such as incoming particles, feed water microorganisms, effects of dynamic conditions are warranted.

Furthermore, effects of predation are largely unknown and potentially important for the formation and stability of granules. The biology within the granules are today in most studies limited to giving names of microorganisms, based on 16S-rRNA. The results obtained from studies of microbial community composition and dynamics in granules at different operational strategies and wastewater compositions give very complex data and it is hard to draw clear conclusions from this on how the different microbial groups, from phylum to species level, affect granule stability and process performance. Nevertheless, full-scale systems are installed and function well and it is possible to control the systems for efficient COD, nitrogen, and phosphorus removal. However, problems with process disturbances such as granule instability occurs and to be able to optimize the process further, the knowledge about the interactions between different microbial groups, and production of EPS and other molecules that contribute to the formation and stability of granules is required. A detailed analysis of sequencing data from different studies with different wastewater composition and process operational modes would give further insight into how this is interconnected with microbial community composition. Future studies of granular metabolism at high resolution using advanced molecular methods such as meta-transcriptomics/proteomics, will tell us the function and activity of the microbes and this would help to provide knowledge about the microbial mechanisms involved for improved process optimization.

## References

[CR1] Ab Halim MH, Nor Anuar A, Azmi SI, Jamal NSA, Wahab NA, Ujang Z, Shraim A, Bob MM (2015). Aerobic sludge granulation at high temperatures for domestic wastewater treatment. Bioresour Technol.

[CR2] Adav SS, Lee D-J (2008). Extraction of extracellular polymeric substances from aerobic granule with compact interior structure. J Hazard Mater.

[CR3] Adav SS, Lee D-J (2008). Single-culture aerobic granules with Acinetobacter calcoaceticus. Appl Microbiol Biotechnol.

[CR4] Adav SS, Lee D-J, Lai JY (2007). Effects of aeration intensity on formation of phenol-fed aerobic granules and extracellular polymeric substances. Appl Microbiol Biotechnol.

[CR5] Adav SS, Lee D-J, Show K-Y, Tay J-H (2008). Aerobic granular sludge: recent advances. Biotechnol Adv.

[CR6] Adav SS, Lee D-J, Tay J-H (2008). Extracellular polymeric substances and structural stability of aerobic granule. Water Res.

[CR7] Aqeel H, Basuvaraj M, Hall M, Neufeld JD, Liss SN (2016). Microbial dynamics and properties of aerobic granules developed in a laboratory-scale sequencing batch reactor with an intermediate filamentous bulking stage. Appl Microbiol Biotechnol.

[CR8] Bao R, Yu S, Shi W, Zhang X, Wang Y (2009). Aerobic granules formation and nutrients removal characteristics in sequencing batch airlift reactor (SBAR) at low temperature. Hazard Mater.

[CR9] Barr JJ, Cook AE, Bond PL (2010). Granule formation mechanisms within an aerobic wastewater system for phosphorus removal. Appl Environ Microbiol.

[CR10] Barr JJ, Slater FR, Fukushima T, Bond PL (2010). Evidence for bacteriophage activity causing community and performance changes in a phosphorus-removal activated sludge. FEMS Microbiol Ecol.

[CR11] Beun JJ, Hendriks A, Loosdrecht van MCM, Morgenroth E, Wilderer PA, Heijnen JJ (1999). Aerobic granulation in a sequencing batch reactor. Water Res.

[CR12] Beun JJ, Heijnen JJ, Van Loosdrecht MCM (2001). N-removal in a granular sludge sequencing batch reactor. Biotechnol Bioeng.

[CR13] Boltz JP, Smets BF, Rittmann BE, van Loosdrecht MCM, Morgenroth E, Daigger GT (2017). From biofilm ecology to reactors: a focused review. Water Sci Technol.

[CR14] Bruus JH, Nielsen PH, Keiding K (1992). On the stability of activated sludge flocs with implications to dewatering. Water Res.

[CR15] Caudan C, Filali A, Spérandio M, Girbal-Neuhauser E (2014). Multiple EPS interactions involved in the cohesion and structure of aerobic granules. Chemosphere.

[CR16] Chen Y-Y, Lee D-J (2015). Effective aerobic granulation: role of seed sludge. J Taiwan Inst Chem Eng.

[CR17] Dangcong P, Bernet N, Delgenes J-P, Moletta R (1999). Aerobic granular sludge—a case report. Water Res.

[CR18] de Kreuk MK, Van Loosdrecht MCM (2004). Selection of slow growing organisms as a means for improving aerobic granular sludge stability. Water Sci Technol.

[CR19] de Kreuk MK, van Loosdrecht MCM (2005). Simultaneous COD, nitrogen, and phosphate removal by aerobic granular sludge. Biotechnol Bioeng.

[CR20] de Kreuk MK, Pronk M, van Loosdrecht MCM (2005). Formation of aerobic granules and conversion processes in an aerobic granular sludge reactor at moderate and low temperatures. Water Res.

[CR21] de Kreuk MK, Kishida N, van Loosdrecht MCM (2007). Aerobic granular sludge—state of the art. Water Sci Technol.

[CR22] de Kreuk MKCP, Hosseini M, Xavier JB, van Loosdrecht MCM (2007). Kinetic model of a granular sludge SBR: influences on nutrient removal. Biotechnol Bioeng.

[CR23] de Kreuk MK, Kishida N, Tsuneda S, van Loosdrecht MCM (2010). Behavior of polymeric substrates in an aerobic granular sludge system. Water Res.

[CR24] Decho AW, Norman RS, Visscher PT (2010). Quorum sensing in natural environments: emerging views from microbial mats. Trends Microbiol.

[CR25] Devlin TR, Oleszkiewicz JA (2018). Cultivation of aerobic granular sludge in continuous flow under various selective pressure. Bioresour Technol.

[CR26] Devlin TR, di Biase A, Kowalski M, Oleszkiewicz JA (2017). Granulation of activated sludge under low hydrodynamic shear and different wastewater characteristics. Bioresour Technol.

[CR27] Dolinšek J, Lagkouvardos I, Wanek W, Wagner M, Daims H (2013) Interactions of nitrifying bacteria and heterotrophs: identification of a *Micavibrio*-like putative predator of *Nitrospira* spp. Appl Environ Microbiol 79(6):2027–2037 10.1128/AEM.03408-12 PMC3592250 23335755

[CR28] Dulekgurgen E, Ovez S, Artan N, Orhon D (2003). Enhanced biological phosphate removal by granular sludge in a sequencing batch reactor. Biotechnol Lett.

[CR29] Fan X-Y, Gao J-F, Pan K-L, Li D-C, Zhang L-F, Wang S-J (2018). Shifts in bacterial community composition and abundance of nitrifiers during aerobic granulation in two nitrifying sequencing batch reactors. Bioresour Technol.

[CR30] Flemming H-C, Wingender J (2010). The biofilm matrix. Nat Rev Microbiol.

[CR31] Franca RDG, Pinheiro HM, van Loosdrecht MCM, Lourenço ND (2018). Stability of aerobic granules during long-term bioreactor operation. Biotechnol Adv.

[CR32] Gao D, Liu L, Liang H, Wu W-M (2011). Aerobic granular sludge: characterization, mechanism of granulation and application to wastewater treatment. Crit Rev Biotechnol.

[CR33] Gao D, Liu L, Liang H, Wu W-M (2011). Comparison of four enhancement strategies for aerobic granulation in sequencing batch reactors. J Hazard Mater.

[CR34] Gonzalez-Gil G, Holliger C (2011). Dynamics of microbial community structure of and enhanced biological phosphorus removal by aerobic granules cultivated on propionate or acetate. Appl Environ Microbiol.

[CR35] Gonzalez-Gil G, Holliger C (2014). Aerobic granules: microbial landscape and architecture, stages, and practical implications. Appl Environ Microbiol.

[CR36] Gonzalez-Martinez A, Muñoz-Palazon B, Rodriguez-Sanchez A, Maza-Márquez P, Mikola A, Gonzalez-Lopez J, Vahala R (2017). Start-up and operation of an aerobic granular sludge system under low working temperature inoculated with cold-adapted activated sludge from Finland. Bioresour Technol.

[CR37] Guimarães LB, Mezzari MP, Daudt GC, da Costa RHR (2017). Microbial pathways of nitrogen removal in aerobic granular sludge treating domestic wastewater. J Chem Technol Biotechnol.

[CR38] Guo F, Zhang SH, Yu X, Wei B (2011). Variations of both bacterial community and extracellular polymers: the inducements of increase of cell hydrophobicity from biofloc to aerobic granule sludge. Bioresour Technol.

[CR39] Hailei W, Guangli Y, Guosheng L, Feng P (2006). A new way to cultivate aerobic granules in the process of papermaking wastewater treatment. Biochem Eng J.

[CR40] He Q, Zhou J, Wang H, Zhang J, Wei L (2016). Microbial population dynamics during sludge granulation in an A/O/A sequencing batch reactor. Bioresour Technol.

[CR41] He Q, Zhang S, Zou Z, Zheng L, Wang H (2016). Unraveling characteristics of simultaneous nitrification, denitrification and phosphorus removal (SNDPR) in an aerobic granular sequencing batch reactor. Bioresour Technol.

[CR42] He Q, Zhang W, Zhang S, Wang H (2017). Enhanced nitrogen removal in an aerobic granular sequencing batch reactor performing simultaneous nitrification, endogenous denitrification and phosphorus removal with low superficial gas velocity. Chem Eng J.

[CR43] Henriet O, Meunier C, Henry P, Mahillon J (2016). Improving phosphorus removal in aerobic granular sludge processes through selective microbial management. Bioresour Technol.

[CR44] Hermansson M (1999). The DLVO theory in microbial adhesion. Colloid Surf B Biointerfaces.

[CR45] Ho K-L, Lin B, Chen Y-Y, Lee D-J (2009) Biodegradation of phenol using *Corynebacterium* sp. DJ1 aerobic granules. Bioresour Technol 100:5051–5055 10.1016/j.biortech.2009.05.050 19540750

[CR46] Huws SA, McBain AJ, Gilbert P (2005). Protozoan grazing and its impact upon population dynamics in biofilm communities. J Appl Microbiol.

[CR47] Jiang B, Liu Y (2012). Roles of ATP-dependent N-acylhomoserine lactones (AHLs) and extracellular polymeric substances (EPSs) in aerobic granulation. Chemosphere.

[CR48] Jiang B, Liu Y (2013). Dependence of structure stability and integrity of aerobic granules on ATP and cell communication. Appl Microbiol Biotechnol.

[CR49] Jiang Y, Shang Y, Wang H, Yang K (2016). Rapid formation and pollutant removal ability of aerobic granules in a sequencing batch airlift reactor at low temperature. Environ Technol.

[CR50] Johnke J, Cohen Y, de Leeuw M, Kushmaro A, Jurkevitch E, Chatzinotas A (2014). Multiple micro-predators controlling bacterial communities in the environment. Curr Opin Biotechnol.

[CR51] Jousset A (2012). Ecological and evolutive implications of bacterial defences against predators. Environ Microbiol.

[CR52] Khan MZ, Mondal PK, Sabir S (2013). Aerobic granulation for wastewater bioremediation: a review. Can J Chem Eng.

[CR53] Kocaturk I, Erguder TH (2016). Influent COD/TAN ratio affects the carbon and nitrogen removal efficiency and stability of aerobic granules. Ecol Eng.

[CR54] Kończak B, Karcz J, Miksch K (2014). Influence of calcium, magnesium, and iron ions on aerobic granulation. Appl Biochem Biotechnol.

[CR55] Lee D-J, Chen Y-Y, Show K-Y, Whiteley CG, Tay J-H (2010). Advances in aerobic granule formation and granule stability in the course of storage and reactor operation. Biotechnol Adv.

[CR56] Lemaire R, Webb RI, Yuan Z (2008). Micro-scale observations of the structure of aerobic microbial granules used for the treatment of nutrient-rich industrial wastewater. ISME J.

[CR57] Lemaire R, Yuan Z, Blackall LL, Crocetti GR (2008b) Microbial distribution of *Accumulibacter* spp. and *Competibacter* spp. in aerobic granules from a lab-scale biological nutrient removal system. Environ Microbiol 10:354–363 10.1111/j.1462-2920.2007.01456.x 18028415

[CR58] Li Y-C, Zhu J-R (2014). Role of N-acyl homoserine lactone (AHL)-based quorum sensing (QS) in aerobic sludge granulation. Appl Microbiol Biotechnol.

[CR59] Li A-j, Yang S-f, Li X-y, Gu J-d (2008). Microbial population dynamics during aerobic sludge granulation at different organic loading rates. Water Res.

[CR60] Li X-M, Liu Q-Q, Yang Q, Guo L, Zeng G-M, Hu J-M, Zheng W (2009). Enhanced aerobic sludge granulation in sequencing batch reactor by Mg2+ augmentation. Bioresour Technol.

[CR61] Li J, Ding L-B, Cai A, Huang G-X, Horn H (2014). Aerobic sludge granulation in a full-scale sequencing batch reactor. Biomed Res Int.

[CR62] Li Y, Hao W, Lv J, Wang Y, Zhong C, Zhu J (2014). The role of N-acyl homoserine lactones in maintaining the stability of aerobic granules. Bioresour Technol.

[CR63] Li Y, Lv J, Zhong C, Hao W, Wang Y, Zhu J (2014). Performance and role of N-acyl-homoserine lactone (AHL)-based quorum sensing (QS) in aerobic granules. J Environ Sci.

[CR64] Li Y, Zou J, Zhang L, Sun J (2014). Aerobic granular sludge for simultaneous accumulation of mineral phosphorus and removal of nitrogen via nitrite in wastewater. Bioresour Technol.

[CR65] Liébana R, Arregui L, Santos A, Murciano A, Marquina D, Serrano S (2016). Unravelling the interactions among microbial populations found in activated sludge during biofilm formation. FEMS Microbiol Ecol.

[CR66] Liébana R, Modin O, Persson F, Wilén B-M (2018) Integration of aerobic granular sludge and membrane bioreactors for wastewater treatment. Crit Rev Biotechnol:1–16. 10.1080/07388551.2017.1414140 10.1080/07388551.2017.1414140 29400086

[CR67] Lin Y, de Kreuk M, van Loosdrecht MCM, Adin A (2010). Characterization of alginate-like exopolysaccharides isolated from aerobic granular sludge in pilot-plant. Water Res.

[CR68] Liu Y, Tay J-H (2002). The essential role of hydrodynamic shear force in the formation of biofilm and granular sludge. Water Res.

[CR69] Liu Y, Tay JH (2004). State of the art of biogranulation technology for wastewater treatment. Biotechnol Adv.

[CR70] Liu Y-Q, Tay J-H (2006). Variable aeration in sequencing batch reactor with aerobic granular sludge. J Biotechnol.

[CR71] Liu Y-Q, Tay J-H (2015). Fast formation of aerobic granules by combining strong hydraulic selection pressure with overstressed organic loading rate. Water Res.

[CR72] Liu Y, Yang SF, Liu QS, Tay JH (2003). The role of cell hydrophobicity in the formation of aerobic granules. Curr Microbiol.

[CR73] Liu Y, Yang S-F, Tay J-H (2004). Improved stability of aerobic granules by selecting slow-growing nitrifying bacteria. J Biotechnol.

[CR74] Liu YQ, Liu Y, Tay JH (2005). Relationship between size and mass transfer resistance in aerobic granules. Lett Appl Microbiol.

[CR75] Liu X-W, Sheng G-P, Yu H-Q (2009). Physicochemical characteristics of microbial granules. Biotechnol Adv.

[CR76] Liu Y-Q, Moy B, Kong Y-H, Tay J-H (2010). Formation, physical characteristics and microbial community structure of aerobic granules in a pilot-scale sequencing batch reactor for real wastewater treatment. Enzym Microb Technol.

[CR77] Liu YQ, Kong YH, Zhang R, Zhang X, Wong FS, Tay JH, Zhu JR, Jiang WJ, Liu WT (2010). Microbial population dynamics of granular aerobic sequencing batch reactors during start-up and steady state periods. Water Sci Technol.

[CR78] Liu X, Sun S, Ma B, Zhang C, Wan C, Lee D-J (2016). Understanding of aerobic granulation enhanced by starvation in the perspective of quorum sensing. Appl Microbiol Biotechnol.

[CR79] Liu Y-Q, Zhang X, Zhang R, Liu W-T, Tay J-H (2016). Effects of hydraulic retention time on aerobic granulation and granule growth kinetics at steady state with a fast start-up strategy. Appl Microbiol Biotechnol.

[CR80] Liu J, Li J, Tao Y, Sellamuthu B, Walsh R (2017). Analysis of bacterial, fungal and archaeal populations from a municipal wastewater treatment plant developing an innovative aerobic granular sludge process. World J Microbiol Biotechnol.

[CR81] Lochmatter S, Holliger C (2014). Optimization of operation conditions for the startup of aerobic granular sludge reactors biologically removing carbon, nitrogen, and phosphorous. Water Res.

[CR82] Lopez-Vazquez CM, Hooijmans CM, Brdjanovic D, Gijzen HJ, van Loosdrecht MCM (2009). Temperature effects on glycogen accumulating organisms. Water Res.

[CR83] Lopez-Vazquez CM, Oehmen A, Hooijmans CM, Brdjanovic D, Gijzen HJ, Yuan Z, van Loosdrecht MCM (2009). Modeling the PAO–GAO competition: effects of carbon source, pH and temperature. Water Res.

[CR84] Lourenço ND, Franca RDG, Moreira MA, Gil FN, Viegas CA, Pinheiro HM (2015). Comparing aerobic granular sludge and flocculent sequencing batch reactor technologies for textile wastewater treatment. Biochem Eng J.

[CR85] Lv J, Wang Y, Zhong C, Li Y, Hao W, Zhu J (2014). The effect of quorum sensing and extracellular proteins on the microbial attachment of aerobic granular activated sludge. Bioresour Technol.

[CR86] Lv J, Wang Y, Zhong C, Li Y, Hao W, Zhu J (2014). The microbial attachment potential and quorum sensing measurement of aerobic granular activated sludge and flocculent activated sludge. Bioresour Technol.

[CR87] Lv Y, Wan C, Lee D-J, Liu X, Tay J-H (2014). Microbial communities of aerobic granules: granulation mechanisms. Bioresour Technol.

[CR88] Martin MO (2002). Predatory prokaryotes: an emerging research opportunity. J Mol Microbiol Biotechnol.

[CR89] Maszenan AM, Liu Y, Ng WJ (2011). Bioremediation of wastewaters with recalcitrant organic compounds and metals by aerobic granules. Biotechnol Adv.

[CR90] Matz C, Kjelleberg S (2005). Off the hook–how bacteria survive protozoan grazing. Trends Microbiol.

[CR91] Matz C, Bergfeld T, Rice SA, Kjelleberg S (2004). Microcolonies, quorum sensing and cytotoxicity determine the survival of *Pseudomonas aeruginosa* biofilms exposed to protozoan grazing. Environ Microbiol.

[CR92] McSwain Sturm BS, Irvine RL (2008). Dissolved oxygen as a key parameter to aerobic granule formation. Water Sci Technol.

[CR93] McSwain BS, Irvine RL, Hausner M, Wilderer PA (2005). Composition and distribution of extracellular polymeric substances in aerobic flocs and granular sludge. Appl Environ Microbiol.

[CR94] Mishima K, Nakamura M (1991). Self-immobilization of aerobic activated sludge—a pilot study of the aerobic upflow sludge blanket process in municipal sewage treatment. Water Sci Technol.

[CR95] Morgenroth E, Sherden T, Loosdrecht van MCM, Heijnen JJ, Wilderer PA (1997). Aerobic granular sludge in a sequencing batch reactor. Water Res.

[CR96] Mosquera-Corral A, de Kreuk MK, Heijnen JJ, van Loosdrecht MCM (2005). Effects of oxygen concentration on N-removal in an aerobic granular sludge reactor. Water Res.

[CR97] Nadell CD, Xavier JB, Foster KR (2009). The sociobiology of biofilms. FEMS Microbiol Rev.

[CR98] Nancharaiah YV, Kiran Kumar Reddy G (2018). Aerobic granular sludge technology: mechanisms of granulation and biotechnological applications. Bioresour Technol.

[CR99] Ni B-J, Xie W-M, Liu S-G, Yu H-Q, Wang Y-Z, Wang G, Dai X-L (2009). Granulation of activated sludge in a pilot-scale sequencing batch reactor for the treatment of low-strength municipal wastewater. Water Res.

[CR100] Olofsson A-C, Zita A, Hermansson M (1998). Floc stability of green-fluorscent-protein-marked bacteria to flocs in activated sludge. Microbiology.

[CR101] Pronk M, de Kreuk MK, de Bruin B, Kamminga P, Kleerebezem R, van Loosdrecht MCM (2015). Full scale performance of the aerobic granular sludge process for sewage treatment. Water Res.

[CR102] Ren T-T, Liu L, Sheng G-P, Liu X-W, Yu H-Q, Zhang M-C, Zhu J-R (2008). Calcium spatial distribution in aerobic granules and its effects on granule structure, strength and bioactivity. Water Res.

[CR103] Ren T-t, Yu H-q, Li X-y (2010). The quorum-sensing effect of aerobic granules on bacterial adhesion, biofilm formation, and sludge granulation. Appl Microbiol Biotechnol.

[CR104] Rocktäschel T, Klarmann C, Helmreich B, Ochoa J, Boisson P, Sørensen KH, Horn H (2013). Comparison of two different anaerobic feeding strategies to establish a stable aerobic granulated sludge bed. Water Res.

[CR105] Rocktäschel T, Klarmann C, Ochoa J, Boisson P, Sørensen K, Horn H (2015). Influence of the granulation grade on the concentration of suspended solids in the effluent of a pilot scale sequencing batch reactor operated with aerobic granular sludge. Sep Purif Technol.

[CR106] Sarma SJ, Tay JH, Chu A (2017). Finding knowledge gaps aerobic granulation technology. Trends Biotechnol.

[CR107] Schwarzenbeck N, Borges JM, Wilderer PA (2005). Treatment of dairy effluents in an aerobic granular sludge sequencing batch reactor. Appl Microbiol Biotechnol.

[CR108] Seviour T, Pijuan M, Nicholson T, Keller Jr, Yuan Z (2009). Gel-forming exopolysaccharides explain basic differences between structures of aerobic sludge granules and floccular sludges. Water Res.

[CR109] Seviour T, Malde AK, Kjelleberg S, Yuan Z, Mark AE (2012). Molecular dynamics unlocks atomic level self-assembly of the exopolysaccharide matrix of water-treatment granular biofilms. Biomacromolecules.

[CR110] Seviour T, Yuan Z, van Loosdrecht MCM, Lin Y (2012). Aerobic sludge granulation: a tale of two polysaccharides?. Water Res.

[CR111] Sheng G-p, Li A-j, Li X-y, Yu H-q (2010). Effects of seed sludge properties and selective biomass discharge on aerobic sludge granulation. Chem Eng J.

[CR112] Show K-Y, Lee D-J, Tay J-H (2012). Aerobic granulation: advances and challenges. Appl Biochem Biotechnol.

[CR113] Song Z, Pan Y, Zhang K, Ren N, Wang A (2010). Effect of seed sludge on characteristics and microbial community of aerobic granular sludge. J Environ Sci.

[CR114] Suarez C, Persson F, Hermansson M (2015). Predation of nitritation–anammox biofilms used for nitrogen removal from wastewater. FEMS Microbiol Ecol.

[CR115] Świątczak P, Cydzik-Kwiatkowska A (2018). Performance and microbial characteristics of biomass in a full-scale aerobic granular sludge wastewater treatment plant. Environ Sci Pollut Res.

[CR116] Szabó E, Hermansson M, Modin O, Persson F, Wilén B-M (2016). Effects of wash-out dynamics on nitrifying bacteria in aerobic granular sludge during start-up at gradually decreased settling time. Water.

[CR117] Szabó E, Liébana R, Hermansson M, Modin O, Persson F, Wilén B-M (2017). Comparison of the bacterial community composition in the granular and the suspended phase of sequencing batch reactors. AMB Express.

[CR118] Szabó E, Liébana R, Hermansson M, Modin O, Persson F, Wilén B-M (2017b) Microbial population dynamics and ecosystem functions of anoxic/aerobic granular sludge in sequencing batch reactors operated at different organic loading rates. Front Microbiol 8 10.3389/fmicb.2017.00770 PMC5410608 28507540

[CR119] Tan CH, Koh KS, Xie C, Tay M, Zhou Y, Williams R, Ng WJ, Rice SA, Kjelleberg S (2014). The role of quorum sensing signalling in EPS production and the assembly of a sludge community into aerobic granules. ISME J.

[CR120] Tan CH, Koh KS, Xie C, Zhang J, Tan XH, Lee GP, Zhou Y, Ng WJ, Rice SA, Kjelleberg S (2015). Community quorum sensing signalling and quenching: microbial granular biofilm assembly. Npj Biofilms Microbiomes.

[CR121] Tay, Liu, Liu (2001). The effects of shear force on the formation, structure and metabolism of aerobic granules. Appl Microbiol Biotechnol.

[CR122] Tay JH, Ivanov V, Pan S, Tay STL (2002). Specific layers in aerobically grown microbial granules. Lett Appl Microbiol.

[CR123] Urbain V, Block JC, Manem J (1993). Bioflocculation in activated sludge: an analytic approach. Water Res.

[CR124] Verawaty M, Pijuan M, Yuan Z, Bond PL (2012). Determining the mechanisms for aerobic granulation from mixed seed of floccular and crushed granules in activated sludge wastewater treatment. Water Res.

[CR125] Verawaty M, Tait S, Pijuan M, Yuan Z, Bond PL (2013). Breakage and growth towards a stable aerobic granule size during the treatment of wastewater. Water Res.

[CR126] Wagner J, Weissbrodt DG, Manguin V, Ribeiro da Costa RH, Morgenroth E, Derlon N (2015). Effect of particulate organic substrate on aerobic granulation and operating conditions of sequencing batch reactors. Water Res.

[CR127] Wan C, Zhang P, Lee D-J, Yang X, Liu X, Sun S, Pan X (2013). Disintegration of aerobic granules: role of second messenger cyclic di-GMP. Bioresour Technol.

[CR128] Wan C, Yang X, Lee D-J, Liu X, Sun S (2014). Partial nitrification using aerobic granule continuous-flow reactor: operations and microbial community. J Taiwan Inst Chem Eng.

[CR129] Wan C, Lee D-J, Yang X, Wang Y, Wang X, Liu X (2015). Calcium precipitate induced aerobic granulation. Bioresour Technol.

[CR130] Wang S, Shi W, Tang T, Wang Y, Zhi L, Lv J, Li J (2017). Function of quorum sensing and cell signaling in the formation of aerobic granular sludge. Rev Environ Sci Biotechnol.

[CR131] Weber SD, Ludwig W, Schleifer KH, Fried J (2007). Microbial composition and structure of aerobic granular sewage biofilms. Appl Environ Microbiol.

[CR132] Wei D, Shi L, Yan T, Zhang G, Wang Y, Du B (2014). Aerobic granules formation and simultaneous nitrogen and phosphorus removal treating high strength ammonia wastewater in sequencing batch reactor. Bioresour Technol.

[CR133] Weissbrodt DG, Lochmatter S, Ebrahimi S, Rossi P, Maillard J, Holliger C (2012). Bacterial selection during the formation of early-stage aerobic granules in wastewater treatment systems operated under wash-out dynamics. Front Microbiol.

[CR134] Weissbrodt DG, Neu TR, Kuhlicke U, Rappaz Y, Holliger C (2013a) Assessment of bacterial and structural dynamics in aerobic granular biofilms. Front Microbiol 4 10.3389/fmicb.2013.00175 PMC3707108 23847600

[CR135] Weissbrodt DG, Schneiter GS, Furbringer JM, Holliger C (2013). Identification of trigger factors selecting for polyphosphate- and glycogen-accumulating organisms in aerobic granular sludge sequencing batch reactors. Water Res.

[CR136] Weissbrodt DG, Shani N, Holliger C (2014). Linking bacterial population dynamics and nutrient removal in the granular sludge biofilm ecosystem engineered for wastewater treatment. FEMS Microbiol Ecol.

[CR137] Wimpenny J, Manz W, Szewzyk U (2000). Heterogeneity in biofilms. FEMS Microbiol Rev.

[CR138] Winkler MKH, Bassin JP, Kleerebezem R, de Bruin LMM, van den Brand TPH, van Loosdrecht MCM (2011). Selective sludge removal in a segregated aerobic granular biomass system as a strategy to control PAO-GAO competition at high temperatures. Water Res.

[CR139] Winkler MKH, Kleerebezem R, Khunjar WO, de Bruin B, van Loosdrecht MCM (2012). Evaluating the solid retention time of bacteria in flocculent and granular sludge. Water Res.

[CR140] Winkler MK, Kleerebezem R, de Bruin LM, Verheijen PJ, Abbas B, Habermacher J, van Loosdrecht MC (2013). Microbial diversity differences within aerobic granular sludge and activated sludge flocs. App Microbiol Biotechnol.

[CR141] Winkler M-KH, Meunier C, Henriet O, Mahillon J, Suárez-Ojeda ME, Del Moro G, De Sanctis M, Di Iaconi C, Weissbrodt DG (2018). An integrative review of granular sludge for the biological removal of nutrients and recalcitrant organic matter from wastewater. Chem Eng J.

[CR142] Wu L-j, Li A-j, Hou B-l, Li M-x (2017). Exogenous addition of cellular extract N-acyl-homoserine-lactones accelerated the granulation of autotrophic nitrifying sludge. Int Biodeterior Biodegrad.

[CR143] Xavier JB, de Kreuk MK, Picioreanu C, van Loosdrecht MCM (2007). Multi-scale individual-based model of microbial and bioconversion dynamics in aerobic granular sludge. Environ Sci Technol.

[CR144] Xiong Y, Liu Y (2013). Importance of extracellular proteins in maintaining structural integrity of aerobic granules. Colloids Surf B: Biointerfaces.

[CR145] Yuan S, Gao M, Ma H, Afzal MZ, Wang Y-K, Wang M, Xu H, Wang S-G and Wang X-H (2017) Qualitatively and quantitatively assessing the aggregation ability of sludge during aerobic granulation process combined XDLVO theory with physicochemical properties. J Environ Sci in press. 10.1016/j.jes.2017.08.015 10.1016/j.jes.2017.08.015 29778148

[CR146] Zhang W, Li C (2016) Exploiting quorum sensing interfering strategies in gram-negative bacteria for the enhancement of environmental applications. Front Microbiol 6 10.3389/fmicb.2015.01535 PMC4705238 26779175

[CR147] Zhang L, Feng X, Zhu N, Chen J (2007). Role of extracellular protein in the formation and stability of aerobic granules. Enzym Microb Technol.

[CR148] Zhang Q, Hu J, Lee D-J (2016). Aerobic granular processes: current research trends. Bioresour Technol.

[CR149] Zhang C, Sun S, Liu X, Wan C, Lee D-J (2017). Influence of operational conditions on the stability of aerobic granules from the perspective of quorum sensing. Environ Sci Pollut Res.

[CR150] Zheng Y-M, Yu H-Q (2007). Determination of the pore size distribution and porosity of aerobic granules using size-exclusion chromatography. Water Res.

[CR151] Zhou D, Niu S, Xiong Y, Yang Y, Dong S (2014). Microbial selection pressure is not a prerequisite for granulation: dynamic granulation and microbial community study in a complete mixing bioreactor. Bioresour Technol.

[CR152] Zita A, Hermansson M (1997). Determination of bacterial cell surface hydrophobicity of single cells in cultures and in wastewater in situ. FEMS Microbiol Lett.

